# Filters comprised of sand and Zero Valent Iron hold promise as tools to mitigate risk posed by *Cyclospora cayetanensis* oocysts

**DOI:** 10.1016/j.fawpar.2024.e00243

**Published:** 2024-08-31

**Authors:** C. Yeager, M. Tucker, A. Gutierrez, C. O'Brien, M. Sharma, V. Fournet, J.P. Dubey, M. Jenkins, K. Kniel, B.M. Rosenthal

**Affiliations:** a Animal Parasitic Diseases Laboratory, Beltsville Agricultural Research Center, North East Area, Agricultural Research Service, United States Department of Agriculture, Beltsville, MD 20705, USA; b Environmental Microbial and Food Safety Laboratory, Beltsville Agricultural Research Center, North East Area, Agricultural Research Service, United States Department of Agriculture, Beltsville, MD 20705, USA; c Department of Animal and Food Science, University of Delaware, Newark, DE 29716, USA

**Keywords:** Cyclospora cayetanensis, Eimeria, Filtration, Zero valent Iron, Water, Irrigation, Mitigation

## Abstract

Irrigation water contaminated by human fecal material may elevate the risk of produce contamination with the enteric parasite *Cyclospora cayetanensis.* Oocysts of *C. cayetanensis* are resistant to commonly used disinfectants and a method of removing *C. cayetanensis* from irrigation water would mitigate this risk. We evaluated zero valent iron (ZVI) sand filtration as one such method. We sought to determine if sand filters containing ZVI outperformed those without ZVI. We first evaluated the abundant poultry parasites *Eimeria maxima*, *E. tenella* and *E. acervulina* as surrogates for *C. cayetanensis*. We determined if a miniaturized gravity fed ZVI-sand filter, scaled to evaluate scarce supplies of *C. cayetanensis* oocysts, provided useful information about the performance of larger filtration systems. Filters were inoculated with oocysts, rinsed, and the resulting filtrate examined microscopically for oocysts. We performed experiments to measure the effect of varying ZVI concentrations, repeated filter use, simulated agricultural water, and oocyst size and condition. We then compared the performance of mini filters to that of larger, gravity-fed pool filters and found that ZVI-sand filtration was far more effective at removing *Eimeria* spp. from water when compared to sand filtration, at both scales. Sand mini filters retained 13–54 % of *E. acervulina* oocysts, and pool filters retained 82 %, but when combined with 50 % (mini filter) or 35 % (pool filter) *v*/v ZVI, mini filters retained 89–99 % of oocysts and pool filters retained >99 %. The effectiveness of the mini filters increased with increasing ZVI concentration, and the addition of ZVI far outweighed the influence of any other measured variable. We then performed experiments including *C. cayetanensis*, which provided similar results to those utilizing *Eimeria*; 59 % of inoculated *C. cayetanensis* oocysts were retained in sand mini filters, and 97 % in mini filters composed of 35 % *v*/v ZVI. In sum, ZVI is highly effective in removing oocysts from water and *Eimeria* is a useful surrogate for *C. cayetanensis* to assess filtration. ZVI-sand filtration shows promise as a tool to mitigate the risk of *C. cayetanensis* contamination of irrigation water. Further studies should evaluate the performance of ZVI-sand in pressurized fast filtration systems under a range of field conditions.

## 1 Introduction

*Cyclospora cayetanensis*, a human gastrointestinal coccidian parasite, contaminates water and produce, threatening food safety (Chacin-Bonilla, 2010; Hadjilouka and Tsaltas, 2020). Direct contact between infected and susceptible individuals likely does not contribute to infection, because oocysts require several days, after excretion, to become infective (Almeria et al., 2019; CDC, 2019). Instead, infection generally occurs when someone ingests water or uncooked produce contaminated with *C. cayetanensis* oocysts. Water contaminated with human fecal material is one potential avenue for crop contamination. *C. cayetanensis* oocysts have been detected in irrigation water (Thurston-Enriquez et al., 2002; Tram et al., 2008; Almeria et al., 2023), wash water (Almeria et al., 2019; Shahatha et al., 2021) and water used for pesticide application (Sathyanarayanan and Ortega, 2004) during produce production, posing a risk for crop contamination.

Countries with robust sanitation systems benefit from reduced environmental contamination with human waste; weaker protections exist for rural, marginalized, or vulnerable communities within these countries and in developing countries (Gasteyer and Vaswani, 2004, Hughes et al., 2005, Chacín-Bonilla et al., 2008, EPA 2021, Mueller and Gasteyer, 2021). Parasitic illness disproportionately affects people in socioeconomically disadvantaged communities worldwide that face barriers to access safe housing, sanitation, and educational resources (Hotez, 2008; Chacín-Bonilla, 2017; Flowers et al., 2019; Chacin-Bonilla and Santin, 2023), and a lack of adequate sanitation increases the risk of fecal organisms contaminating water. In the United States 65–80 million people, including 60 % of rural households, use onsite wastewater treatment options such as septic systems (American Society of Civil Engineers, 2024). People that cannot afford the expense of installing, maintaining or replacing these systems, up to 67 % of the homes in some counties, resort to using straight pipes that discharge untreated wastewater into yards or ditches and contaminate surface water. Communities plagued by wastewater pollution from failed septic systems or straight pipe discharge have high rates of parasitic illness (Flowers et al., 2019; Maxcy-Brown et al., 2021), as high as 62.8 % in one community (Singer et al., 2020). This lack of sanitation in agricultural areas increases the risk of cyclosporiasis and other food-borne illnesses (Dawson, 2005; Tefera et al., 2018), and the risk is only expected to increase as the climate warms and extreme weather events and flooding become more commonplace (Kistemann et al., 2002; Cooper et al., 2016; Trtanj et al., 2016; Ziska et al., 2016; Pozio, 2020; Semenza and Paz, 2021)*.*

The environmentally resistant oocyst stage of *C. cayetanensis* likely survives in soil and water for prolonged intervals and pre-harvest mitigation strategies can reduce the resultant risk. Few studies have defined environmental distribution and persistence of *C. cayetanensis* in real-world conditions (Almeria et al., 2023). However, Naganathan et al. (2022) found in a meta-analysis of 33 studies worldwide that over 17 % of irrigation water samples contained *C. cayetanensis*, Onstad et al. (2019) detected *C. cayetanensis* in soil, water and animal feces in a region near Chicago impacted by combined sewer outfalls, Sathyanarayanan and Ortega (2006) found that *C. cayetanensis* can survive on experimentally contaminated basil leaves for at least a week, and Chacin-Bonilla (2010) found that, in endemic areas, risk of infection increased with poor sanitation, contact with soil or animals, and contaminated food and water. Extended survival of *C. cayetanensis* in agricultural environments increases the risk of crop contamination, and oocysts of related coccidian parasites have been shown to persist and remain infections for months to years. Species of *Eimeria* retain their ability to infect their natural hosts after years of storage in laboratory conditions (Jenkins et al., 2023) and oocysts of *Toxoplasma gondii* have proved infectious after residing 18 months in the soil (Frenkel et al., 1975) and at least 2 years in seawater (Lindsay and Dubey, 2009). Knowledge of the prevalence of cyclosporiasis is limited and is complicated by a lack of surveillance programs and the existence of asymptomatic infection. As many as 86 % of infections detected in a surveillance study of a population experiencing recurrent *C. cayetanensis* transmission were asymptomatic (Chacín-Bonilla et al., 2007), and in areas considered non-endemic *C. cayetanensis* has repeatedly been found in surface water and wastewater suggesting undetected infections (Chacin-Bonilla and Santin, 2023). The potential for persistent environmental contamination with oocysts of *C. cayetanensis* highlights the need for mitigation strategies capable of reducing the likelihood that such oocysts will contaminate produce. To this end, some growers have instituted significant safety and sanitation programs intended to limit risk such as improvements to water quality and enforcement of hygienic practices like handwashing, but *C. cayetanensis* resists disinfectant treatment of water and produce (Ortega et al., 2008; FDA, 2022). Where fecal contamination of surface and groundwater occurs, producers need an affordable way to remove oocysts from irrigation and wash water, prevent contamination of produce, and reduce the risk of cyclosporiasis in farm workers and consumers.

A simple filter comprised of sand and zero valent iron (ZVI) holds promise as a method of preventing *C. cayetanensis* from contaminating produce. Sand filters are easily maintained devices made of inexpensive and readily available materials (Elbana et al., 2012; Nyberg et al., 2014; Majsztrik et al., 2017) that growers use to improve the quality of irrigation water and prevent clogging of irrigation lines in fields (Ingram et al., 2012; Kim et al., 2020; Wang et al., 2022). Zero valent iron (ZVI), a byproduct of industrial activities (Satapanajaru et al., 2006; Tellen et al., 2010), is an inexpensive and widely available material that, when added to sand filters adsorbs to and dramatically reduces bacterial (Ingram et al., 2012; Kulkarni et al., 2020; Zhu et al., 2023) and viral (Shearer and Kniel, 2018; Chopyk et al., 2019) pathogens in filtered water. ZVI-sand filtration of artificially contaminated irrigation water reduces contamination of leafy greens by *Escherichia coli* (Anderson-Coughlin et al., 2021; Kim et al., 2021) and *Listeria* spp. (Marik et al., 2019).

The benefits of ZVI as a component of sand filters have not yet been fully evaluated with parasites, but preliminary data suggest that large ZVI-sand filters reduce their levels substantially (Kniel, 2019). Tests evaluating ZVI-sand filtration of bacteria in irrigation water have typically employed large filters with pore volumes (the total volume of voids contained within a granular material) of several liters; such tests often filter tens or hundreds of liters, and experimental conditions simulate agricultural water, (Marik et al., 2019; Kim et al., 2020; Anderson-Coughlin et al., 2021). At this scale, unattainably large numbers of *C. cayetanensis* oocysts would be needed to obtain reliable estimates of filter performance. *C*. *cayetanensis* oocysts are difficult to obtain because clinical samples from infected patients constitute their sole source; clinics do not routinely test for *C. cayetanensis,* and many patients recover without treatment or diagnosis (CDC, 2019; Tucker et al., 2022). No means yet exist to propagate *C*. *cayetanensis* in vitro or in any laboratory animal, and *C. cayetanensis* in the laboratory poses a health risk for laboratory personnel because only a few oocysts can cause an infection. Fortunately, species of *Eimeria* share an especially close phylogenetic relationship to *C. cayetanensis* (Relman et al., 1996) and therefore share many physical attributes, elevating them as a logical surrogate to test many biological properties, including filter performance (Kniel et al., 2007; Tucker et al., 2021; Dubey et al., 2022; Tucker et al., 2022; Augendre et al., 2023). *Eimeria* spp. include parasites of chickens, which produce oocysts by the millions, and pose no health risk to humans.

Here, we sought to determine if ZVI-sand filtration removes coccidian parasites such as *Eimeria* and *C. cayetanensis* from water more effectively than sand alone, if *Eimeria* spp. are acceptable surrogates for *C. cayetanensis* regarding ZVI-sand filtration, and if small scale filtration experiments can provide insight into the dynamics of larger systems. To do so, we evaluated filter performance at a variety of scales in filters of varying compositions. We performed experiments using a 24 ml “mini filter” scale, described here for the first time, that allows precise estimates of filter performance while requiring 50–100 times fewer oocysts than do larger filter systems. Mini filters enabled us to perform experiments directly on *C. cayetanensis* and validate the relevancy of *Eimeria* data on the responses of *C. cayetanensis* to risk mitigation via filtration*.*

## 2 Materials and methods

### 2.1 Animal experimentation

Animal experiments were performed following the protocol (22–06) approved by the BARC Institutional Animal Use and Care Committee, United States Department of Agriculture. Chickens utilized in this study exhibited no outward signs of severe disease over the course of the study. After the study's conclusion, all chickens were humanely euthanized; all efforts were made to minimize animal suffering.

### 2.2 Parasites

#### 2.2.1 Species of parasites used

In addition to experiments using *Cyclospora cayetanensis,* we employed three species of *Eimeria*: *E. acervulina* (18 × 15 μm) is the *Eimeria* species closest in size to *C. cayetanensis* (10 μm) and has been used as a surrogate for *C. cayetanensis* for produce decontamination (Lee and Lee, 2001; Kniel et al., 2007); this species was used in the large-scale filtration experiment described below. *E. tenella* (22 × 19 μm) has been compared to *C. cayetanensis* with respect to metabolism (Liu et al., 2016) and filtration (Kniel, 2019), and was used in this work to develop and test the mini filters described below. *E. maxima* (31 × 21 μm) is easily distinguished from *E. tenella*, *E. acervulina* and *C. cayetanensis* by size and, used in combination with the smaller species as an internal standard, can be used to determine the effects of oocyst size on removal by filtration.

#### 2.2.2 Propagation of Eimeria parasites and preparation of oocysts for use in experiments

*Eimeria* oocysts used here were obtained as described before in Ryley et al. (1976) and Jenkins et al. (2009). Briefly, oocysts were separated from feces from experimentally infected chickens via saturated NaCl floatation and centrifugation. The recovered oocysts were preserved in 2 % potassium dichromate, sporulated at 29 °C for 1–3 days and then stored at 4 °C. We separated oocysts from the potassium dichromate via centrifugation (Jouan CR422 centrifuge at 3309 x*g* for 5 min) and except where specified, we resuspended them in 2 ml 6 % sodium hypochlorite in a 15 ml centrifuge tube on a rocker (24 rpm) at room temperature (RT) for 15 min. We then separated oocysts from the bleach via centrifugation (Jouan CR422 centrifuge at 3309 x*g* for 5 min) and resuspended in deionized (DI) water. We repeated the centrifugation and resuspension steps three times and stored the cleaned oocysts at 4 °C until use. Such bleach treatment reduces bacterial and fungal contamination of *Eimeria* oocysts. and removes the outer oocyst wall without affecting the viability of *Eimeria* oocysts. The outer wall (acid-fast lipids and glycoproteins) differs in composition from the inner wall (β-1,3-glucan fibrils and glycoproteins) (Bushkin et al., 2013). To determine whether such differences influence their response to filtration, we compared bleached and unbleached oocyst passage through sand and ZVI-sand filters (here and in Supplementary Data 1). We used unbleached oocysts in experiments using agricultural water or *C. cayetanensis* oocysts, to best mimic environmental conditions.

#### 2.2.3 Preparation of C. cayetanensis oocysts for use in experiments

*C. cayetanensis* oocysts used in this work originated from anonymized clinical samples provided by the Mayo Clinic, Rochester, Minnesota. Oocysts, preserved in EcoFix for 1 year and stored at 4 °C, were removed from the clinical samples via sucrose floatation and centrifugation. Samples were centrifuged (Thermo Scientific Sorvall Legend XTR) at 1962 x g for 5 min and the fixative was removed. Pellets were resuspended in 0.5 ml DI water and 0.75 % *w*/*v* of Alconox and gently shaken by hand to dislodge oocysts from large particles in the clinical samples. Next, 2 ml of 2 M sucrose was added and the samples were centrifuged for 10 min at 1363 x*g*. Oocysts were allowed to float undisturbed for 10 min and the supernatant was transferred to a new 15 ml centrifuge tube and diluted to 15 ml with DI water. Oocysts were centrifuged at 2671 x*g* for 5 min; the supernatant was removed, and the oocysts were resuspended in 3 ml PBS and stored at 4 °C until use. To determine if fixative treatment or age affected filtration of oocysts, we compared fresh, fixed and old unbleached *Eimeria* oocysts (Supplementary Data 2).

#### 2.2.4 Preparation of inoculum

*Eimeria* oocyst concentrations were determined with an Improved Neubauer hemocytometer. Oocysts/ml was calculated using the formula oocysts/ml = (oocysts per square) * 10,000 and this value was used to calculate the volume of stock needed for each inoculum. The target inoculum level for *Eimeria* spp. experiments ranged from 1000 to 80,000 oocysts per 6 ml inoculum. *C. cayetanensis* oocysts removed from PBS and resuspended in DI water were enumerated with a McMasters chamber and combined with *E. maxima* or *E. acervulina.* Pairing species in a filter provides an internal standard and enables direct evaluation of species- or size-specific variation in filter efficiency. The target level for combined *Eimeria* and *C. cayetanensis* inocula ranged from 8000 to 12,000*C. cayetanensis* and 11,000 to 20,000 *Eimeria* oocysts per 6 ml inoculum. For each experiment, typically comprising 6 filters, a bulk inoculum was subdivided into 6.3 ml aliquots. A 300 μl sample was taken from each aliquot and enumerated; the average of these re-tested estimates defined the inoculum level for each experiment. One large-scale experiment with a pool filter was conducted. A target number of 15 million *E. acervulina* oocysts were suspended in 50 ml DI water, counted four times with a hemacytometer to define the inoculum level, and then diluted to 10 l with municipal water.

### 2.3 Mini filter construction

**Fig. 1:**
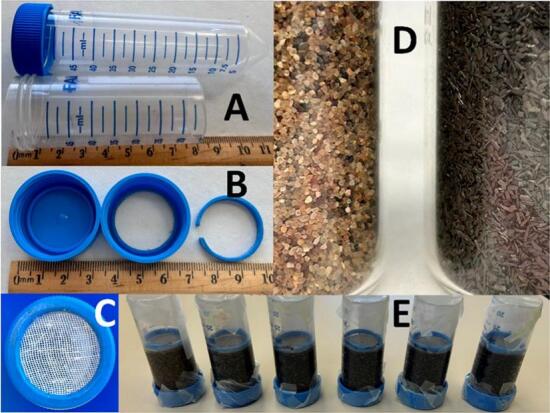
Construction of mini filters. A - Intact and cut centrifuge tubes. B left to right - Intact centrifuge cap, cap with center removed, retaining ‘gasket’. C - End view of assembled tube, gauze and cap. D left to right - Sand and ZVI used for filtration medium. E - Assembled mini filters loaded with oocysts and capped with parafilm for a 24-h incubation.

We constructed mini filters from 50 ml disposable plastic centrifuge tubes (Fig. 1). The bottom 3 cm of each tube was removed with a Dremel MultiPro rotary cutting tool (Fig. 1A). We removed the center of the cap as far as the sealing ring to allow for water drainage and used the sealing ring of a second cap to make a ‘gasket’ for the top of the filter (Fig. 1B). Two 3.75 cm circles were cut out of fine weave bleached cotton gauze (500 μm pore size). One was trapped between the cap and the threaded portion of the tube to form the bottom of the filter. (Fig. 1C). Filters were filled to a 5 cm depth with 24.5 ml of either sand or a sand/ZVI mixture (Fig. 1D) and tapped to settle the medium. The second gauze circle was trapped between the tube and the gasket and pushed down to ensure the filtration medium was not mixed or disturbed during filtration (Fig. 1E). A vial was put inside the finished filter and used to tamp down the medium until packed well.

Filtration medium was made of various *v*/v combinations of sand (Fig. 1D, left) (U.C. 1.6 filter sand, Northern Filter Media, grain size 0.45–0.55 mm, 1.58 g/ml) and zero valent iron (aggregate from Peerless Metal Powder and Abrasives, grain size 0.43–0.60 mm, 2.92 g/ml) (Fig. 1D right). For ZVI-sand filters we weighed sand and ZVI, combined them in a container and shook well until homogeneous before we filled the filters. The porosity (volume of void space per volume of sand or ZVI-sand mixture) is 35 % for 100 % sand and 46 % for a 1:1 v/v mixture of ZVI and sand. Porosity was determined by measuring the volume of water that saturates 100 ml of filter media (Gutierrez, unpublished data). For all ZVI-sand filters, the relative contribution of ZVI to the total filter is based on volume. Each filter contains 24.54 ml of filtration medium (5 cm depth), thus a 100 % sand filter contained 38.77 g sand (24.54 ml * 1.58 g/ml) and had a pore volume of 8.6 ml (24.54 ml sand * 0.35), and a 50 % ZVI-sand filter contained 19.39 g sand (12.27 ml * 1.58 g/ml) and 35.83 g ZVI (12.27 ml * 2.92 g/ml) and had a pore volume of 11.3 ml (24.54 ml * 0.46).

### 2.4 General procedure for mini filter experiments

#### 2.4.1 Inoculation and filtration

The general procedure for all mini filter gravity filtration experiments was as follows. Each mini filter test included three steps: prerinse, inoculation and filtration. Filters were prerinsed by pouring 60 ml DI water into the open top of the filters immediately before use. This rinse, containing fine particulates from the sand and ZVI was discarded. Filters were inoculated with premeasured aliquots of *Eimeria* or *C. cayetanensis* oocysts suspended in 6 ml deionized (DI) water and poured into the open tops of the filters. Filtrate released during inoculation was collected and evaluated for oocyst concentration. The inoculated oocysts were allowed to remain in the wet filters for 1 min or, in extended exposure experiments, the filters were capped with parafilm (Fig. 1E) and stored at 4 °C for 24 h. We then filtered 60 ml DI water divided into 5 pre-measured 12 ml aliquots that were sequentially poured into the open top of the filters. Filtrate released after each inoculation or filtration step was collected with funnels into individual 15 ml centrifuge tubes. Each aliquot was then evaluated separately for oocyst concentration.

#### 2.4.2 Concentration and enumeration of filtrate

Filtrate samples were concentrated to a volume that allowed enumeration (minimum volume = 300 μl, maximum concentratio*n* = 4000 oocysts/ml) employing a McMasters chamber, with requisite statistical precision. Oocysts in filtrate became obscured by ZVI aggregates after centrifugation, so we adapted a gravity settling procedure used for environmental water samples (Anderson and Karlson, 2017) that recovered oocyst numbers comparable to those yielded by centrifugation, but with less aggregation. We stored filtrate samples upright in 15 ml centrifuge tubes at 4 °C for at least 12 h to allow all oocysts to settle to the conical portion of the tube. We carefully removed the supernatant, not below the beginning of the conical portion of the tube, and counted the concentrated sample or (if further concentration was desired) we transferred the samples to individual 2 ml low retention microcentrifuge tubes, allowed them to settle upright and undisturbed for at least 3 h, and then removed additional supernatant and counted the sample. All *Eimeria* samples were mixed 1:1 with 2 M sucrose and enumerated in a McMasters chamber at 100× final magnification. All oocysts were counted regardless of sporulation status as the filtration efficiency of sporulated and unsporulated oocysts was the same (unpublished data, ANOVA *p* > 0.05). The oocyst concentration was calculated with the formula: oocysts/ml = ((# oocysts in 6 lanes) * 2) / 0.15 and the total oocysts in each filtrate aliquot was calculated with the formula: oocysts in filtrate = oocysts/ml * (volume filtrate collected / volume concentrated filtrate). *C. cayetanensis* oocysts did not float well in the standard 1:1 ratio of sample: 2 M sucrose used in McMasters chambers, resulting in undercounts; therefore, we used a 1:4 sample: 2 M sucrose ratio for all McMasters counts involving *C. cayetanensis*. We counted these smaller oocysts at a final magnification of 200×.

We compared the gravity settling concentration method to concentration via centrifugation by suspending 1500 *E. tenella* oocysts in 4 ml DI and then concentrating them by either settling or by centrifugation (Jouan CR422 centrifuge at 3309 x*g* for 5 min in 15 ml centrifuge tubes when volumes were > 1.5 ml and Eppendorf 5425R centrifuge at 6010 x*g* for 4 min in 1.5 ml microcentrifuge tubes for smaller volumes). We recovered comparable proportions of the inoculum after either centrifugation or settling (80 % vs 85 %, *n* = 4 for each method, *p* > 0.05 ANOVA).

### 2.5 Filter experiments (summarized in Table 1)

#### 2.5.1 Filter scale: Pool filter versus mini filter

We conducted a large-scale test using a pool filter (Intex Krystal Clear Sand Filter Pump, model no. SF90110–2) that contained 8.2 l of either 100 % sand (porosity = 35 %, pore volume = 2.87 l) or a 35 % ZVI/65 % sand mixture (ZVI-35, porosity = 44 %, pore volume = 3.61 l) to compare the behavior of mini filters to that of larger systems. A 50 l tank with an outlet mounted 11 in. above the filter input supplied water to the pool filter through a 1 ½ inch diameter plastic hose. This allowed water to pass through the filter via gravity, as in the mini filters. The flow rate through the filter, measured with a stopwatch as the tank drained, was determined to be 9.7 l/min (SD 1.5 l/min, *n* = 9). Pool filters were preconditioned with >150 l municipal water prior to use. We performed the same three steps with the pool filters as we did with the mini filters, namely prerinse, inoculation, and filtration, but added a 50 l municipal water rinse between each of three successive filtration runs with the same filter. A 250 ml sample was taken prior to each inoculation to verify that no oocysts from earlier inoculations still exited the filter; none were found. Filters were prerinsed with 50 l municipal water, inoculated with 1.25 × 10^7^ to 1.41 × 10^7^ *E. acervulina* oocysts suspended in 10 l municipal water, and then 6 l municipal water was added to the filter. All water exiting the filter from the point of inoculation until the end of filtration, 18.6 l, was collected in a 20 l carboy. The filtrate was stirred, and three 250 ml samples were obtained. Each 250 ml sample was concentrated to 50 ml (sand filters) or 600 μl (ZVI-35 filters) and examined individually for oocysts as previously described. We compared the results of the pool filter to that of sand and 50 % ZVI/50 % sand *v*/v mixture (ZVI-50) mini filters inoculated with 6300–15,600 *E. acervulina* oocysts and run using the standard procedure presented in Section 2.4.

**Table 1:** Description of mini filter experiments, including treatment type, filter type, number of oocysts per 6 ml inoculum and number of replicates per treatment. ZVI-XX signifies an XX% *v*/v ZVI/sand filter.

Experiment	Treatment	Filter type	*E. maxima* # oocysts *n* replicates	*E. tenella* # oocysts *n* replicates	*E. acervulina* # oocysts *n* replicates	*C. cayetanensis* # oocysts *n* replicates
Filter scale: Pool filter versus mini filter	Pool	Sand			12.5–14 million ***3***	
	Pool	ZVI-35			12.5–14 million ***3***	
	Mini	Sand			6300–15,600 ***6***	
	Mini	ZVI-50			6300–15,600 ***6***	
Detection of low and high inoculum levels in filtrate	<20,000 oocysts	Sand		6500–14,700 ***9***		
	<20,000 oocysts	ZVI-50		960–14,700 ***19***		
	>20,000 oocysts	Sand		31,700-81,300 ***7***		
	>20,000 oocysts	ZVI-50		20,700-81,300 ***12***		
Percent ZVI: 0 %–50 % v/v ZVI	ZVI five 10 % increments	Sand, ZVI-10, 20, 30, 40, 50		80,000-82,000 ***3***		
Species filtration and release time	No ZVI	Sand	6600 ***3***	67,000-82,000 ***4***	6300 ***3***	
	50 % ZVI	ZVI-50	6600 ***3***	67,000-82,000 ***7***	6300 ***3***	
Filter reuse: 5 repeated rinse-filter cycles	1–5 cycles	Sand	7500 ***3***	6500 ***3***		
	1–5 cycles	ZVI-50	7500 ***3***	6500 ***3***		
Water type: DI versus simulated agricultural water (unbleached oocysts)	DI	Sand		13,600 ***3***		
	Agricultural water	Sand		13,600 ***3***		
	DI	ZVI-50		13,600 ***3***		
	Agricultural water	ZVI-50		13,600 ***3***		
*Eimeria* spp. comparison with *C. cayetanensis* (unbleached oocysts)	No ZVI	Sand	15,200-57,400 ***10***	17,200-33,300 ***6***	9100–16,200 ***9***	7550–11,600 ***4***
	35 % ZVI	ZVI-35	22,000 ***1***		13,700 ***3***	7550-11,600 ***4***
	50 % ZVI	ZVI-50	15,200-57,400 ***6***	33,300 ***3***	9100–16,200 ***6***	

#### 2.5.2 Detection of low and high inoculum levels in filtrate

Any filtration experiment conducted on *C. cayetanensis* would be characterized by a low inoculum level. We therefore examined the effect of low inoculum levels on mini filter performance by comparing low inoculum (<20,000 oocysts) to high inoculum (>20,000 oocysts) levels to ensure proportional oocyst recovery in each case. We inoculated sand and ZVI-50 filters with as few as 960 and as many as 81,000 oocysts of *E. tenella.* We conducted these experiments according to the standard protocol.

#### 2.5.3 Percent ZVI: 0 %–50 % ZVI/sand v/v

We performed experiments evaluating the effect of varied ZVI concentrations on filtration at each 12 ml filtration aliquot. We prepared three filters each with ZVI v/v concentrations of 0 %, 10 %, 20 %, 30 %, 40 % and 50 %, inoculating each filter with 81,000 bleached oocysts of *E. tenella*. We capped the filters with parafilm and incubated them at 4 °C for 24 h. We then rinsed each incubated filter, collected filtrate and concentrated the samples by the standard procedure described above.

#### 2.5.4 Species filtration and release time

We performed experiments to determine if oocyst size affected the volume of filtrate needed to release oocysts from sand and ZVI-50 mini filters. We inoculated filters with 6600 *E. maxima*, 67,000–82,000 *E. tenella* or 6300 *E. acervulina* oocysts, rinsed and analyzed filtrate using the standard procedure described above.

#### 2.5.5 Filter reuse: 5 repeated rinse-filter cycles

In another experiment we subjected triplicate sand, and ZVI-50 filters, to five repeated filtration cycles. In each cycle, the filter was prerinsed with 60 ml DI water, inoculated with 7500 *E. maxima* and 6500 *E. tenella* in 6 ml DI water, retained in the filter for 1 min, and then 60 ml DI was passed through the filter.

#### 2.5.6 Water type: DI versus simulated agricultural water

We tested the effect of water composition on filter efficiency using the recipe for test agricultural water presented in the FDA's revised efficacy protocol for reduction of foodborne bacteria in agricultural water for preharvest (Blackburn, 2020). This water contains 1.6 g/l sea salt and 10 mg/l humic acid. All agricultural water was adjusted to pH 6.5. Six sand and six ZVI-50 filters were inoculated with 13,600 unbleached *E. tenella* oocysts and treated according to the standard procedure, except that for three of each type of filter the DI water was replaced (at all steps) with simulated agricultural water.

#### 2.5.7 Microscopic evaluation of ZVI adsorption to the oocyst wall

The utility of ZVI addition to sand filters lies mainly in its strong adsorptive properties, which can retain oocysts within a filter and reduce their contamination of filtered water. We therefore examined the adsorption of ZVI to the oocyst wall of *E. tenella* via microscopy. Bleached and unbleached oocysts used for microphotography were exposed to ZVI for 24 h. The ZVI was then rinsed with DI water and the filtrate was concentrated via gravity settling. Oocysts were then photographed with a Leica DM750 compound microscope fitted with a Path4K camera or processed as in (Vieira et al., 2023) and imaged on a Hitachi SU7000 cryo-scanning electron microscope.

#### 2.5.8 Eimeria spp. comparison with C. cayetanensis

Replicate sand and ZVI-50 filters were inoculated with unbleached *E. maxima* (*n =* 9 sand, *n* = 6 ZVI-50), *E. tenella* (*n* = 6 sand, *n* = 3 ZVI-50) or *E. acervulina* (*n* = 3 sand and ZVI-50). Additionally, four sand and four ZVI-35 filters were inoculated with unbleached *E. maxima* (22,000 oocysts *n* = 1) or *E. acervulina* (7800 oocysts, *n* = 3) paired with 7550–11,600*C. cayetanensis* oocysts. ZVI-35 filters were chosen for experiments involving *C. cayetanensis* to improve oocyst recovery and enumeration. Each inoculum was mixed, sampled, and filtered in the same way as for single species experiments and analyzed according to the standard protocol.

### 2.6 Statistical tests

Statistical analysis of experimental results was performed in Excel. We performed ANOVA analyses and regression analyses with the XL toolbox NG add-in. The significance value was set to *p* < 0.05 and experiments with significant differences were analyzed with pairwise students-*t-*tests.

## 3 Results

### 3.1 Filter experiments

#### 3.1.1 Filter scale: Pool filter versus mini filter

**Fig. 2:**
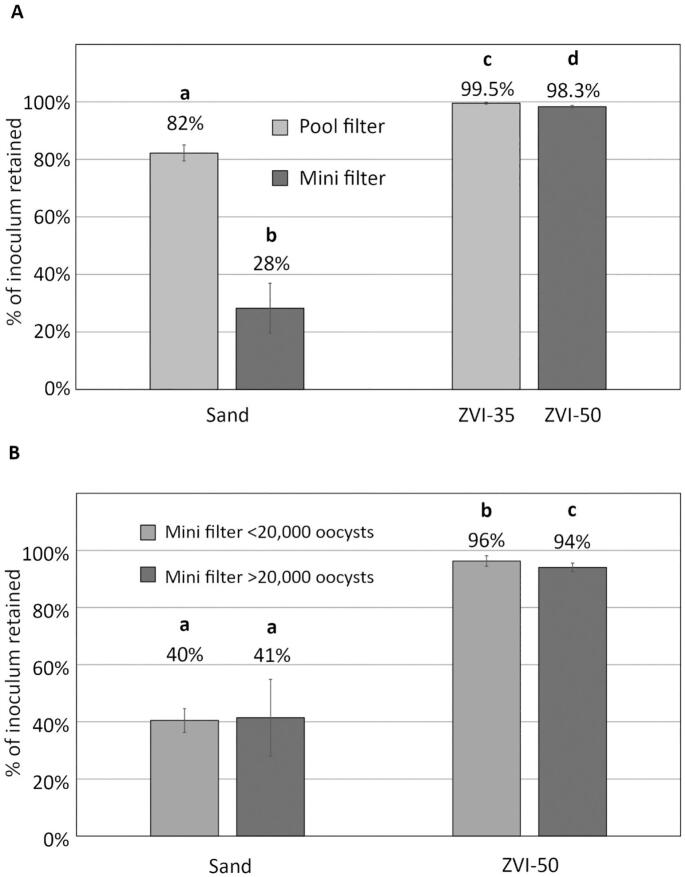
Panel A: Percent of inoculated *E. acervulina* oocysts retained by sand and ZVI-50 mini filters and sand and ZVI-35 pool filters. Panel B: Percent of oocysts retained by sand and ZVI-50 mini filters inoculated with low and high levels of *E. tenella*. Error bars represent +/− 1 SD. Bars with the same letter are not significantly different (ANOVA, 2 tailed t-test *p* > 0.05).

Sand mini filters retained 28 % of *E. acervulina*; far larger sand pool filters performed significantly better, trapping 82 % (Fig. 2A). At each scale, adding ZVI to sand filters improved their performance dramatically. ZVI-50 mini filters retained 98.3 % of *E. acervulina* oocysts, while ZVI-35 pool filters retained 99.55 %. Thus, adding 50 % ZVI increased the performance of mini filters by 42-fold; adding 35 % ZVI increased the performance of pool filters by 36-fold.

#### 3.1.2 Detection of low and high inoculum levels in filtrate

In sand filters, we found no significant performance difference in mini filters inoculated with many (>20,000) or few (〈<20,000) oocysts (Fig. 2B). By contrast, significantly more oocysts were retained by ZVI-50 filters inoculated with low oocyst concentrations, and the oocyst level in filtrate approached, but did not cross, the lower limit of oocysts enumerable with statistical precision. For ZVI-50 filters, the standard error was 7.2 % of the mean for high oocyst concentrations and 11 % of the mean for low concentrations.

#### 3.1.3 Percent ZVI: 0 % - 50 % *v*/v ZVI

**Fig. 3:**
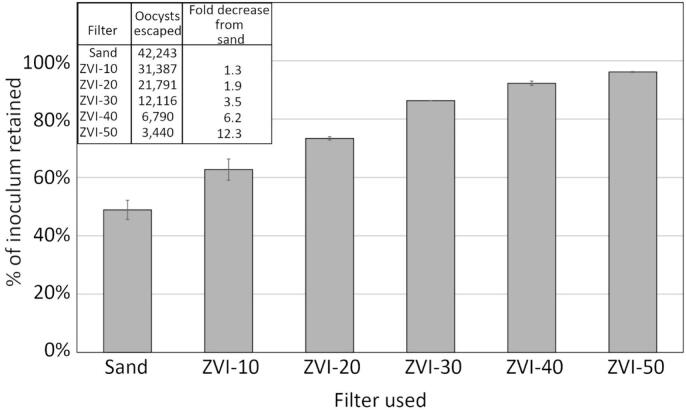
Percent of 80,000–82,000 bleached *E. tenella* oocysts retained by mini filters containing 0 %–50 % ZVI. Error bars represent +/− 1 SD. All values are significantly different (ANOVA, 2-tailed *t*-test, *p* < 0.05).

Filter performance steadily increased with increasing ZVI concentration (Fig. 3). Merely 10 % ZVI improved filter performance 1.3 times over sand alone; 50 % ZVI improved performance over 12-fold.

#### 3.1.4 Species filtration and release time

**Fig. 4:**
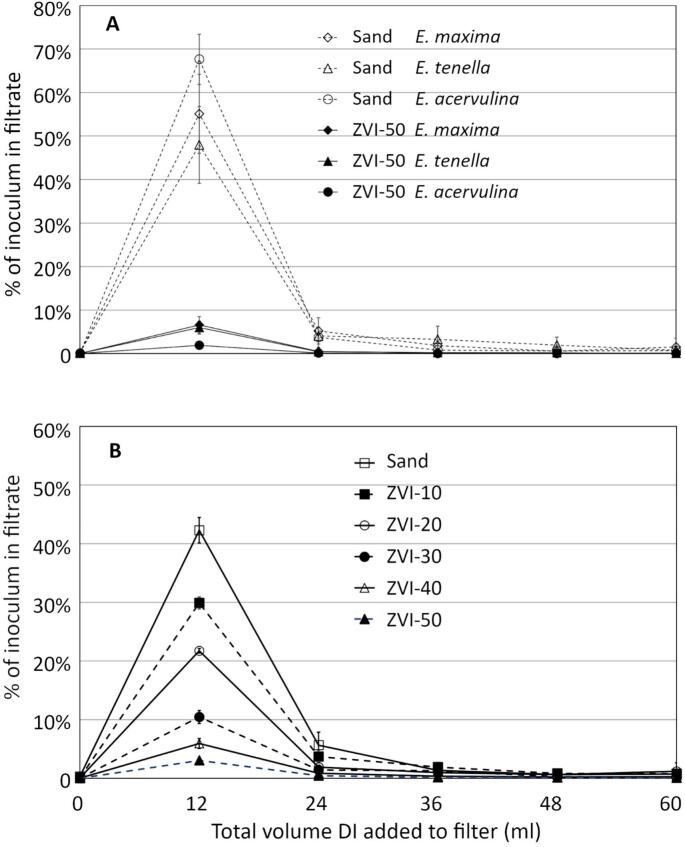
Panel A: Recovery of *E. maxima*, *E. tenella* or *E. acervulina* oocysts in successive 12 ml filtrate aliquots from sand and ZVI-50 mini filters. The measurement at 0 ml represents the oocysts collected in the 6 ml released from filters during inoculation. Panel B: Recovery of *E. tenella* in successive 12 ml filtrate aliquots from mini filters containing a range of ZVI concentrations presented in Fig. 3. Error bars represent +/− 1 SD. All points at the first 12 ml aliquot are significantly different from adjacent points at 0 ml and 24 ml (ANOVA, 2-tailed t-test, *p* < 0.05).

We evaluated whether the timing of oocyst emergence from mini filters varied with ZVI composition or *Eimeria* species. In every case, regardless of species or ZVI concentration, the first 12 ml rinse (about one pore volume) carried the most oocysts (Fig. 4A, B), consistent with prior observations in larger filters (unpublished data).

#### 3.1.5 Filter reuse: 5 repeated rinse-filter cycles

**Fig. 5:**
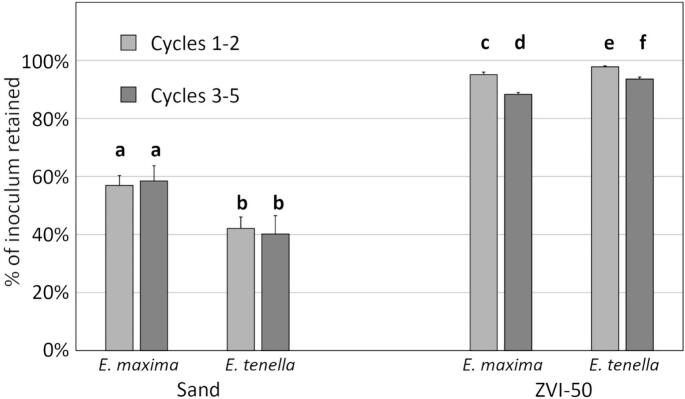
Percent of *E. maxima* and *E. tenella* retained by sand and ZVI-50 mini filters subjected to five rinse-inoculate-filter cycles. The first 12 ml of filtrate was enumerated. Error bars represent +/− 1 SD, *n* *=* 3. Bars with the same letter are not significantly different (ANOVA, 2-tailed t-test, *p* > 0.05).

Sand filters showed consistent performance throughout the five rinse-inoculate-filter cycles for both *E. maxima* and *E. tenella*, but the ZVI-50 filters began releasing more oocysts after cycle 2 (Fig. 5), going from 95 % to 88 % of *E. maxima* and from 98 % to 94 % of *E. acervulina* oocysts retained. Throughout the five cycles, ZVI-50 filters retained far more oocysts than did 100 % sand filters (40–58 %).

#### 3.1.6 Water type: DI versus simulated agricultural water

**Fig. 6:**
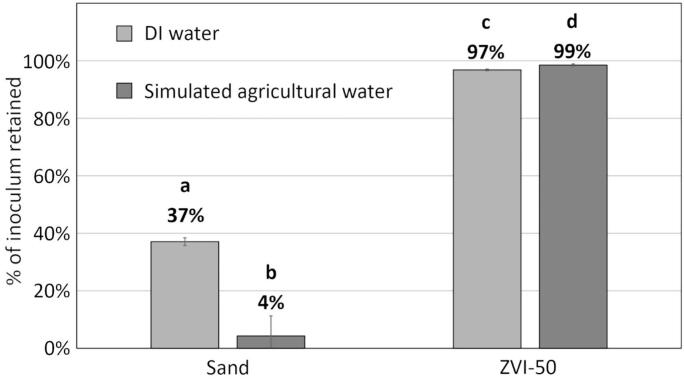
Percent of unbleached *E. tenella* oocysts retained by sand and ZVI-50 mini filters when DI water or simulated agricultural water was used. Bars with the same letter are not significantly different (ANOVA, 2-tailed t-test, *p* > 0.05). Error bars represent +/− 1 SD, *n* *=* 3.

We then evaluated mini filter performance employing simulated agricultural water, understanding that typical irrigation water contains dissolved salts and organic material absent from DI water. Notably, almost no unbleached oocysts were retained by sand filters in the simulated agricultural water treatment (Fig. 6). By contrast, ZVI-50 mini filter performance increased slightly when using agricultural water as compared to DI water (ANOVA, 2-tailed t-test, *p* < 0.05).

#### 3.1.7 Microscopic evaluation of ZVI adsorption to the oocyst wall

**Fig. 7:**
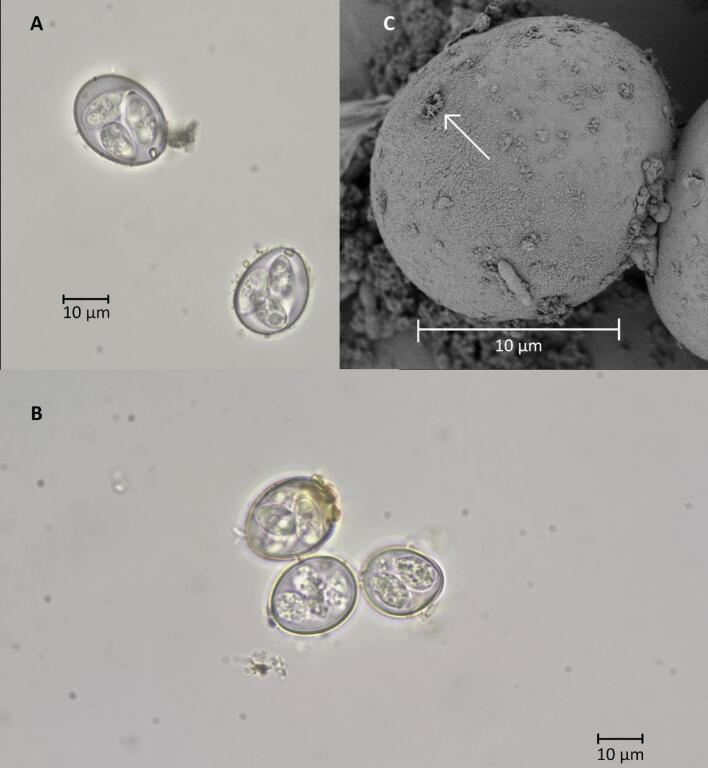
ZVI adsorbs strongly to both bleached and unbleached oocysts, even after sucrose floatation and rinsing. Panels A,B: Light micrographs (100×) of iron attached to the surface of bleached (Panel A) and unbleached (Panel B) *E. tenella* oocysts. Panel C: Iron particles (arrow) attached to an unbleached *E. acervulina* oocyst.

Microscopic examination revealed that ZVI particles remained adsorbed to bleached and unbleached *Eimeria* oocysts exposed to ZVI-50 filters (Fig. 7). We observed this even in oocysts separated from ZVI-50 filters with sucrose floatation that had undergone multiple rinses as described in Supplementary Data 3, indicating strong attachment to the oocyst wall.

#### 3.1.8 Eimeria spp. comparison with C. Cayetanensis

**Fig. 8:**
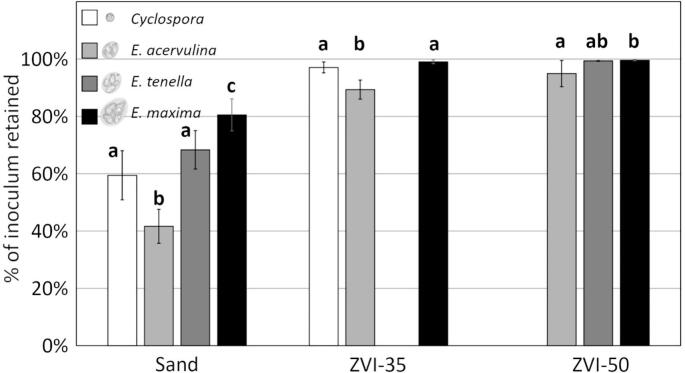
unbleached *Eimeria* and *C. cayetanensis* oocysts retained by sand, ZVI-35 and ZVI-50 mini filters. Bars within one filter group with the same letter are not significantly different (ANOVA, 2-tailed t-test, *p* < 0.05). Error bars indicate +/− 1 SD.

Finally, we compared filter performance for three species of *Eimeria* against that for *C. cayetanensis*, finding that sand and ZVI-35 filters performed similarly for unbleached oocysts of *C. cayetanensis* and *Eimeria* (Fig. 8). Among the *Eimeria* species, larger oocysts were retained more than smaller oocysts in all filters tested. Sand filters retained 81 % of *E. maxima*, the largest species, and 68 % and 42 % of *E. tenella* and *E. acervulina* respectively. ZVI-50 filters retained 99.6 % of *E. maxima*, 99 % of *E. tenella* and 95 % of *E. acervulina*. *C. cayetanensis*, the smallest oocyst examined, was retained more than *E. acervulina*, the smallest *Eimeria* species tested. Sand filters retained 59 % of *C. cayetanensis* oocysts and ZVI-35 filters retained 97 % of *C. cayetanensis* but only 89 % of *E. acervulina*. A caveat that bears repeating is that the *C. cayetanensis* available for our use had been fixed during clinical diagnosis of human cases. Subsequent tests of fixed and unfixed *Eimeria* (Table SD-1) suggested only limited impact of short-term fixation on filter performance.

### 3.2 Sand and 50 % ZVI filter efficiency across a wide range of inoculum concentration

**Fig. 9:**
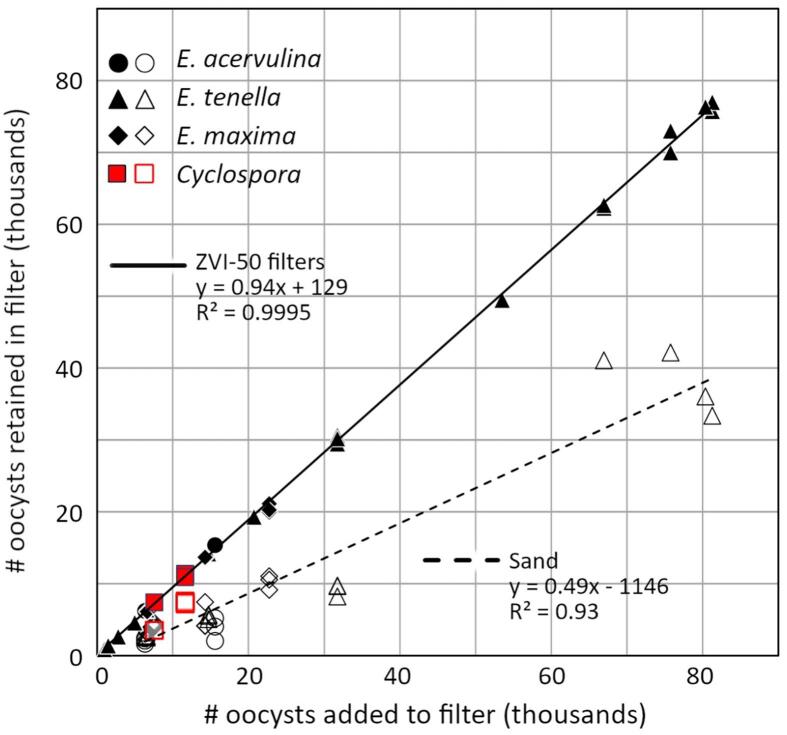
Retention of bleached *Eimeria* oocysts by mini filters inoculated with a range of oocyst levels from 960 to 82,000. Open symbols designate sand filters; closed symbols designate ZVI-50 filters. All results represent the oocysts observed in the first 12 ml aliquot rinse. The slopes and intercepts of the regression lines presented are significantly different (*p* < 0.05). Results from four sand and four ZVI-35 filters inoculated with unbleached *C. cayetanensis* oocysts are presented for comparison and fall within the range predicted by *Eimeria* surrogates. Sand *n* *=* 37, ZVI *n* *=* 52.

As detailed above, we executed several independent experiments to explore, in isolation, the effects of inoculum concentration, ZVI concentration, water composition, filter reuse, oocyst size, bleach treatment, fixation effects and oocyst age on filter efficiency. Taken together, we collected data from a total of 37 sand and 52 ZVI-50 mini filters that were inoculated with bleached oocysts of *E. tenella, E. maxima or E. acervulina* and treated according to the standard protocol (Table 1)*.* Many of these served as controls for other experimental treatments. We compiled results from these experiments and estimated, by means of linear regression, the relationship between the number of oocysts inoculated onto a filter and the number of oocysts retained by the filter (Fig. 9). There was a strong positive correlation between oocysts inoculated and retained for sand filters, regardless of parasite species (r^2^ = 0.93). This relationship was even less variable in ZVI-50 filters (r^2^ = 0.9995). Irrespective of inoculum size, about half of all bleached oocysts were retained by sand filters (average 42 %, SD 12 %, *n* = 37). By contrast, over 95 % of oocysts were retained by ZVI-50 filters (average 95.3 %, SD 2.5 %, *n* = 52). Sand filters did not outperform ZVI-50 filters in any mini filter test; the maximum percentage of bleached oocysts retained by a sand filter was 62 % and the minimum percentage retained by ZVI-50 filters was 88 %.

**Fig. 10:**
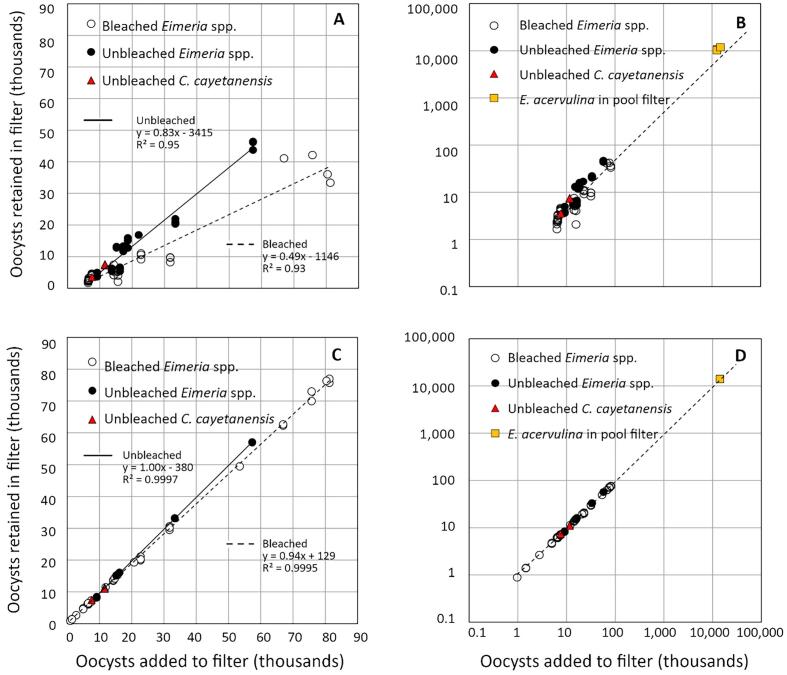
Retention of three species of bleached and unbleached *Eimeria* oocysts from sand and ZVI-50 mini filters. Unbleached *C. cayetanensis* oocysts (red) and bleached *E. acervulina* in pool filters (yellow) are included for comparison but were not included in the regression analysis. 10 A: Retention of bleached (open symbols) and unbleached (closed symbols) *Eimeria* oocysts by sand mini filters. 10B: Sand mini filter performance over 4 orders of magnitude. Log scale allows visualization of experiments differing vastly in inoculum size. The regression line for bleached oocysts from 10 A is included for comparison. 10C: Retention of three species of bleached (open symbols) and unbleached (closed symbols) *Eimeria* oocysts by ZVI-50 mini filters. 10D: ZVI filter performance scales over 4 orders of magnitude. Log scale allows visualization of experiments differing vastly in inoculum size. The regression line for bleached oocysts from 10C is included for comparison. The slopes and intercepts of all regression lines are significantly different (*p* < 0.05). (For interpretation of the references to colour in this figure legend, the reader is referred to the web version of this article.)

The results of experiments using bleached oocysts proved conservative when compared to experiments employing unbleached oocysts, as would be those found in the environment. Fig. 10 presents the combined results of 89 tests performed on bleached *Eimeria* oocysts and 37 tests performed on unbleached oocysts. Four tests performed on unbleached *C. cayetanensis* are included for comparison. Both sand (Fig. 10A) and ZVI-50 (Fig. 10C) filters retained more unbleached than bleached oocysts, and there was again a strong positive correlation between inoculated and retained oocysts (sand r^2^ = 0.95, ZVI-50 r^2^ = 0.9997). An average of 64 % (SD 18 %, *n* = 25) of unbleached *Eimeria* were retained by sand and 98 % (SD 4 %, *n* = 15) by ZVI-50 filters. Unbleached *C. cayetanensis*, inoculated on sand and 35 % ZVI filters, behaved comparably to unbleached *Eimeria*, further supporting the use of *Eimeria* as surrogates for *C. cayetanensis* with respect to filtration tests. A log scale presentation of the same data, including the results of pool filter experiments (Fig. 10B, Fig. 10D), shows that mini filters provide a good representation of the results gained from gravity fed filters on a larger scale.

## 4 Discussion

We sought to determine if filters comprised of sand alone, or sand plus ZVI, impede passage of *Eimeria* and *C. cayetanensis* oocysts. We found that species of *Eimeria* proved useful surrogates when evaluating the efficacy of such filters against *Cyclospora cayetanensis*, and we demonstrated that results obtained from mini filters provide useful information about the performance of larger filtration systems. Under all conditions tested, adding ZVI to a sand filter dramatically increased retention of *Eimeria* and *C. cayetanensis* oocysts; even addition of 10 % ZVI (by volume) to a sand filter caused a significant performance improvement (Fig. 3). In fact, ZVI is such an effective amendment to sand filters that tests comparing *C. cayetanensis* to *Eimeria* required development of a small-scale filter enabling meaningful experiments using few oocysts. Varying the inoculum of *Eimeria* in mini filters yielded proportional results, justifying tests employing necessarily limited numbers of *C. cayetanensis* oocysts. Increased retention when oocysts were most scarce (Fig. 2B) suggests that our tests approached the minimum usable inoculum level, even at this small scale. Even then, ZVI addition strongly improved filter performance. Inoculum levels and oocyst retention (in sand and ZVI-50 filters) retained a robust proportional relationship across a wide range of inoculum levels (Fig. 9, Fig. 10). We then used *E. acervulina* as a surrogate for *C. cayetanensis* to demonstrate the utility of ZVI as an additive to sand filters on a larger scale and found, at both scales, that ZVI-sand filters removed almost all oocysts from the filtrate. ZVI adsorbs to the oocyst wall (Fig. 7), and we conclude that such adsorption drives enhanced filter performance provided by ZVI.

The results of the pool filter experiment are subject to several limitations. First, pool filters were rinsed and reused between inoculations. While we did not detect breakthrough oocysts in the rinsewater between filtration experiments, it is reasonable to suggest that some oocysts from an earlier inoculation could travel through the filter and be collected in a subsequent experiment. Such delayed oocyst release would decrease the measured efficiency of the pool filters in subsequent runs, so our experiments provide a conservative estimate of pool filter performance. Second, the oocysts used in the pool filter experiment were bleached (to reduce bacterial and fungal contamination), and this treatment alters the characteristics of the oocyst wall and the interaction between oocysts and ZVI. Bleaching did affect filtration in each type of filter, unbleached oocysts being retained more strongly than bleached oocysts (Fig. 10, Fig. SD-1). Given that bleaching worsened filter performance, such experiments again provide conservative estimates of filter performance, and we recommend that unbleached oocysts be used to validate future filtration systems. Finally, most of our experiments used either DI water or municipal water for filtration, unlike water typically used for irrigation. Simulated agricultural water decreased the performance of sand filters but increased the performance of ZVI-50 filters. Humic acid may explain reduced performance of sand filters (Wang et al., 2022) because humic acid competes with bacteria for deposition sites in sand filters (Yang et al., 2012) and may do the same with oocysts. Increased performance of ZVI-50 filters may have been caused by pH or the presence of salts, which increases *Cryptosporidium* retention in sand filters (Kim et al., 2010). In any event, the better performance of ZVI filters using simulated agricultural water suggests that filters applied in real-world situations may outperform predictions derived from tests using deionized water.

These data attest to a strong interaction between coccidian parasites, such as *C. cayetanensis*, and ZVI and indicate that gravity fed filtration, similar to that found in a slow sand filtration system (Huisman and Wood, 1974) removes most such parasites from the water column. The fluid drag force exerted on bound particles is much higher in a pressurized fast filtration system than in a gravity fed system and lowers the retention of particulates (Torkzaban et al., 2007) including *Cryptosporidium* (Kim et al., 2010) and bacteria (Wang et al., 2022). The performance of ZVI-sand filters over time, especially in pressurized systems, merits further examination to fully understand their promise and limitations under a range of conceivable field applications.

We conclude that gravity fed filters comprised of ZVI and sand are highly effective in removing the coccidian parasites *Eimeria* spp. and *C. cayetanensis* from water, that *Eimeria* is an appropriate surrogate for *C. cayetanensis* in sand and ZVI filters, and that such surrogates enable testing of large filtration systems akin to those used by some growers to purify irrigation water. Such systems would be especially valuable in areas that suffer from poor water quality and an elevated risk of *C. cayetanensis* contamination. The mini filters enable efficient, replicated experimental evaluation of several parameters at a scale that conserves resources, avoids environmental risks inherent to large-scale field experiments and yields valuable data without endangering the health of laboratory personnel. In sum, gravity fed filters comprised of 50 % sand and 50 % ZVI serve as a powerful tool to remove almost all coccidian parasites, be they *Eimeria* or *C. cayetanensis* and do so to a far greater extent than do filters comprised only of sand*.*

## CRediT authorship contribution statement

**C. Yeager:** Conceptualization, Methodology, Investigation, Writing – original draft, Visualization. **M. Tucker:** Conceptualization, Methodology, Investigation, Writing – review & editing, Supervision. **A. Gutierrez:** Methodology, Resources. **C. O'Brien:** Investigation, Resources, Supervision. **M. Sharma:** Conceptualization, Methodology, Resources, Writing – review & editing, Supervision, Funding acquisition. **V. Fournet:** Methodology. **J.P. Dubey:** Conceptualization, Supervision, Funding acquisition. **M. Jenkins:** Conceptualization, Resources, Writing – review & editing, Funding acquisition. **K. Kniel:** Conceptualization, Writing – review & editing, Funding acquisition. **B.M. Rosenthal:** Conceptualization, Methodology, Investigation, Resources, Writing – original draft, Supervision, Project administration, Funding acquisition.

## Declaration of competing interest

The authors declare the following financial interests/personal relationships which may be considered as potential competing interests:

Benjamin Rosenthal reports financial support was provided by Center For Produce Safety. If there are other authors, they declare that they have no known competing financial interests or personal relationships that could have appeared to influence the work reported in this paper.

## Appendix A Supplementary data

Figure SD1 Interaction between bleach status and oocyst size. Image 1

Table SD1 Filtration of fresh, fixed and old unbleached *Eimeria* oocysts. Image 1

Figure SD2 Recovery of large numbers of filter-exposed *Eimeria* oocysts for physiological experiments. Image 1
