# Supplementary material for: Filters comprised of sand and Zero Valent Iron hold promise as tools to mitigate risk posed by Cyclospora cayetanensis oocysts
Source: Food Waterborne Parasitol. 2024 Aug 31;37:e00243. doi: 10.1016/j.fawpar.2024.e00243 (PMC11409009; doi:10.1016/j.fawpar.2024.e00243)
Supplement: Supplementary file 1 — Figure SD1 Interaction between bleach status and oocyst size. [file mmc1.docx]

Supplementary data 1 - Interaction between bleach status and oocyst size:

It is reasonable to assume that bleach treatment will affect the filtration of Eimeria oocysts. Figure S-1A presents the results from filtration experiments performed on unbleached oocysts of *E. acervulina* (sand n = 9, ZVI-50 n=6), *E. tenella* (sand n=6, ZVI-50 n=3) and *E. maxima* (sand n=10, ZVI-50 n=6) conducted as in the standard procedure. Figure S1-B presents results performed on bleached oocysts of *E. acervulina* (sand n = 6, ZVI-50 n=6), *E. tenella* (sand n=16, ZVI-50 n=31) and *E. maxima* (sand n=15, ZVI-50 n=15). ANOVA analysis revealed a significant effect of both oocyst size and bleach status on filtration, as well as a significant interaction between the two (p<0.05). Large unbleached oocysts were retained by sand filters more than smaller oocysts. The same was true for bleached oocysts inoculated on sand filters, but there was an inverse relationship between size and oocyst retention when bleached oocysts were inoculated on ZVI-50 filters. We hypothesize that small bleached oocysts with a high surface area to volume ratio adsorb to ZVI-50 filters more strongly than do larger bleached oocysts.


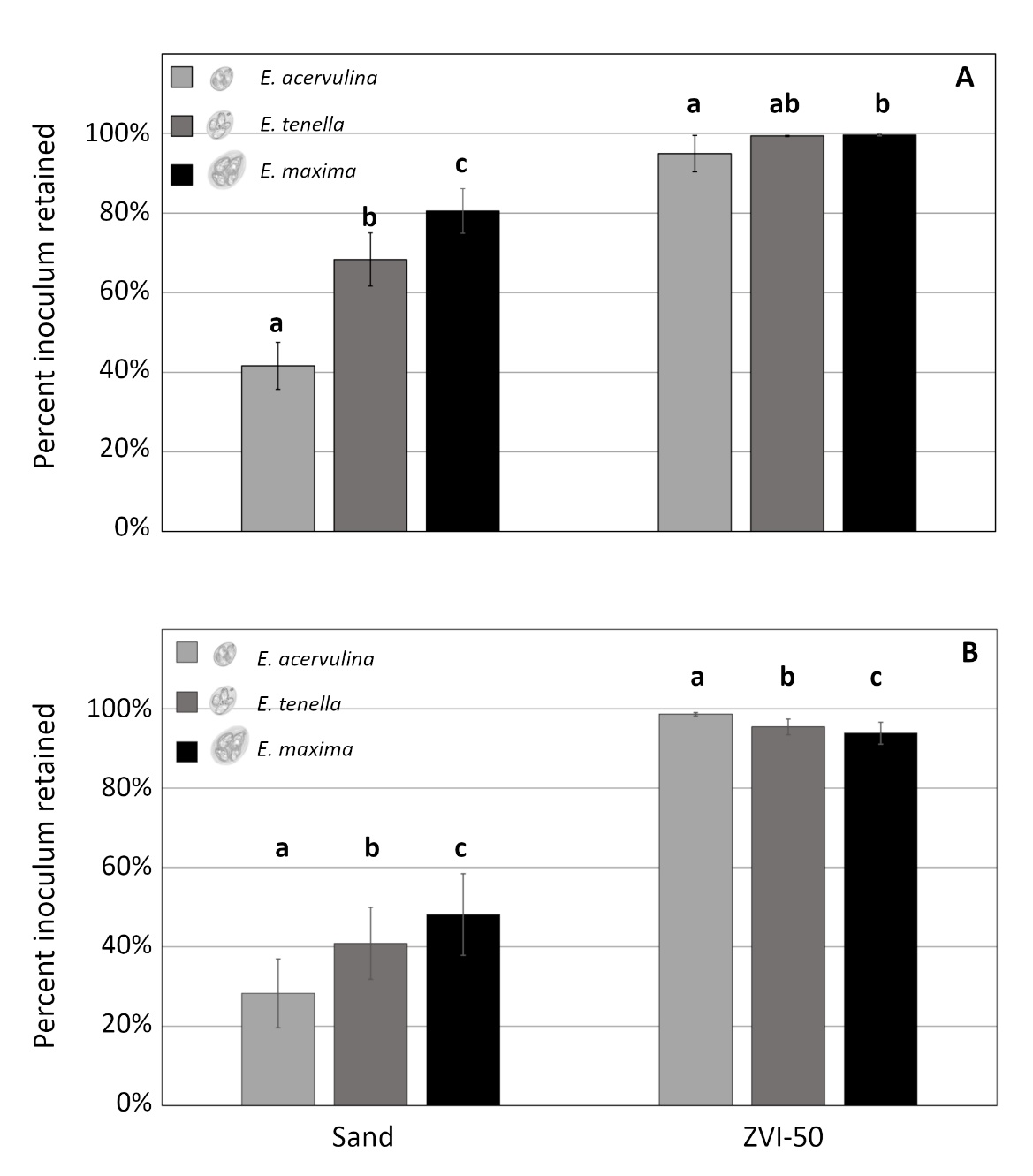


**Figure SD-1:** Retention of unbleached (**Panel A**) and bleached (**Panel B**) *E. acervulina*, *E. tenella* and *E. maxima* oocysts in sand and ZVI-50 filters. Values with the same letter within one filter type are not significantly different (ANOVA, 2-tailed t-test, *p*<0.05). Error bars indicate +/- 1 SD.
