# Supplementary material for: Filters comprised of sand and Zero Valent Iron hold promise as tools to mitigate risk posed by Cyclospora cayetanensis oocysts
Source: Food Waterborne Parasitol. 2024 Aug 31;37:e00243. doi: 10.1016/j.fawpar.2024.e00243 (PMC11409009; doi:10.1016/j.fawpar.2024.e00243)
Supplement: Supplementary file 2 — Table SD1 Filtration of fresh, fixed and old unbleached Eimeria oocysts. [file mmc2.docx]

Supplementary data 2 - Filtration of fresh, fixed and old unbleached Eimeria oocysts:

Oocysts of *C*. cayetanensis used here had been preserved in a zinc sulfate formalin free fixative for more than a year prior to filtration experiments; therefore, we examined the effect of fixation and aging on oocyst filtration. A sample of unbleached *E. acervulina* was preserved in a zinc sulfate formalin free fixative containing 25% ethanol, 7.9% zinc sulfate, 4.8% acetic acid, 1.9% glycerin, 1% methanol, 1% isopropanol and 58.4% DI water, pH 2.4. This sample was stored for 1 week at 4°C and used to evaluate the effect of fixation on filtration efficiency. A separate sample of unbleached oocysts were stored unfixed in DI water for comparison. To test the effects of aging on filtration, we tested oocysts of *E. acervulina* that had been stored at 4 °C in potassium dichromate for greater than three years. Each treatment, unfixed, fixed and old, was inoculated on triplicate sand and ZVI-50 filters according to the standard protocol. The inoculum level used (7,370-9,730 oocysts in 6 ml) was similar to that used for *C*. cayetanensis (7,550-11,600 oocysts in 6 ml). Any effects of aging were too small to achieve statistical significance (2-tailed t-test, *p*>0.05), and were overwhelmed by the strong effects of adding ZVI. We conclude that the effect of ZVI addition in tests involving *C*. *cayetanensi*s minimized any effect of oocyst age and fixation.

**Table SD-1:** Retention of fresh, fixed, and old *E. acervulina* oocysts from triplicate sand and ZVI-sand filters. Values within a filter type (sand or ZVI-50) are not significantly different (ANOVA, 2-tailed t-test, *p*>0.05).

|  | Sand | | ZVI-50 | |
| --- | --- | --- | --- | --- |
| Oocyst type | Mean | SD | Mean | SD |
| Unbleached | 45 % | 7.8 % | 90.9 % | 1.8 % |
| Fixed | 50 % | 5.2 % | 93.7 % | 1.5 % |
| Old | 53 % | 9.6 % | 92.9 % | 0.5 % |
