# Supplementary material for: Filters comprised of sand and Zero Valent Iron hold promise as tools to mitigate risk posed by Cyclospora cayetanensis oocysts
Source: Food Waterborne Parasitol. 2024 Aug 31;37:e00243. doi: 10.1016/j.fawpar.2024.e00243 (PMC11409009; doi:10.1016/j.fawpar.2024.e00243)
Supplement: Supplementary file 3 — Figure SD2 Recovery of large numbers of filter-exposed Eimeria oocysts for physiological experiments. [file mmc3.docx]

***Supplementary data 3 – Recovery of large numbers of filter-exposed Eimeria oocysts for physiological experiments:***

## We used mini filters to expose *Eimeria* oocysts to sand and ZVI-50 as a means of collecting large numbers of oocysts. These oocysts were used to examine the physiological effects of ZVI on Eimeria. We recovered large numbers of oocysts from sand and ZVI filters employing a sucrose floatation method. We inoculated 14.6-28.7 million bleached *E. acervulina* oocysts suspended in DI water onto three sand and six ZVI-50 filters, capped the tubes with parafilm and incubated them 24 hours at 4°C. We then emptied the contents of the filter, 6 ml DI and 17 ml 2M sucrose into a flask. The average volume of water retained by the filter was previously determined by weight and the volumes presented here result in a 1M sucrose concentration. Oocysts were allowed to float, with occasional gentle swirling, for 5 minutes and then allowed to rest undisturbed for 5 minutes. We decanted the supernatant into a 50 ml centrifuge tube and then combined the sand or sand/ZVI with 11.5 ml each DI water and 2M sucrose again to recover additional oocysts. We removed ZVI dust via centrifugation (Jouan CR422 centrifuge at 3309 x g for 5 minutes) and then diluted the supernatant at least 1:7 with DI water. We then concentrated the oocysts down to a 1 ml volume via centrifugation, and when transferring to smaller containers we rinsed the centrifuge bottles and tubes with water to dilute the sucrose and ensure a maximum number of oocysts were recovered. This process enabled recovery of 48% of inoculated *E. acervulina* oocysts from ZVI-50 filters (Figure SI-2). We also inoculated six sand and six ZVI-50 filters with 6,300-15,600 bleached *E. acervulina* oocysts and collected them from filtrate using the general procedure for mini filter experiments. Nearly half of oocysts inoculated on ZVI-50 filters were recovered with the sucrose floatation method, compared to less than 2% collected in filtrate (Figure SI-2). Oocyst recovery from sand filters after sucrose floatation was lower than that from ZVI-50 filters (Figure SI-2) and numerous free sporocysts were observed (unpublished data), suggesting that oocysts in a sand-sucrose suspension were more likely to rupture during mixing than those in ZVI-sand-sucrose. We conclude that sucrose floatation of Eimeria oocysts from ZVI-50 filters is an effective method for retrieving large numbers of ZVI exposed oocysts for further analysis, but filtration is a more effective method of retrieving oocysts from sand filters. Results of experiments utilizing these oocysts will be presented in a later study.


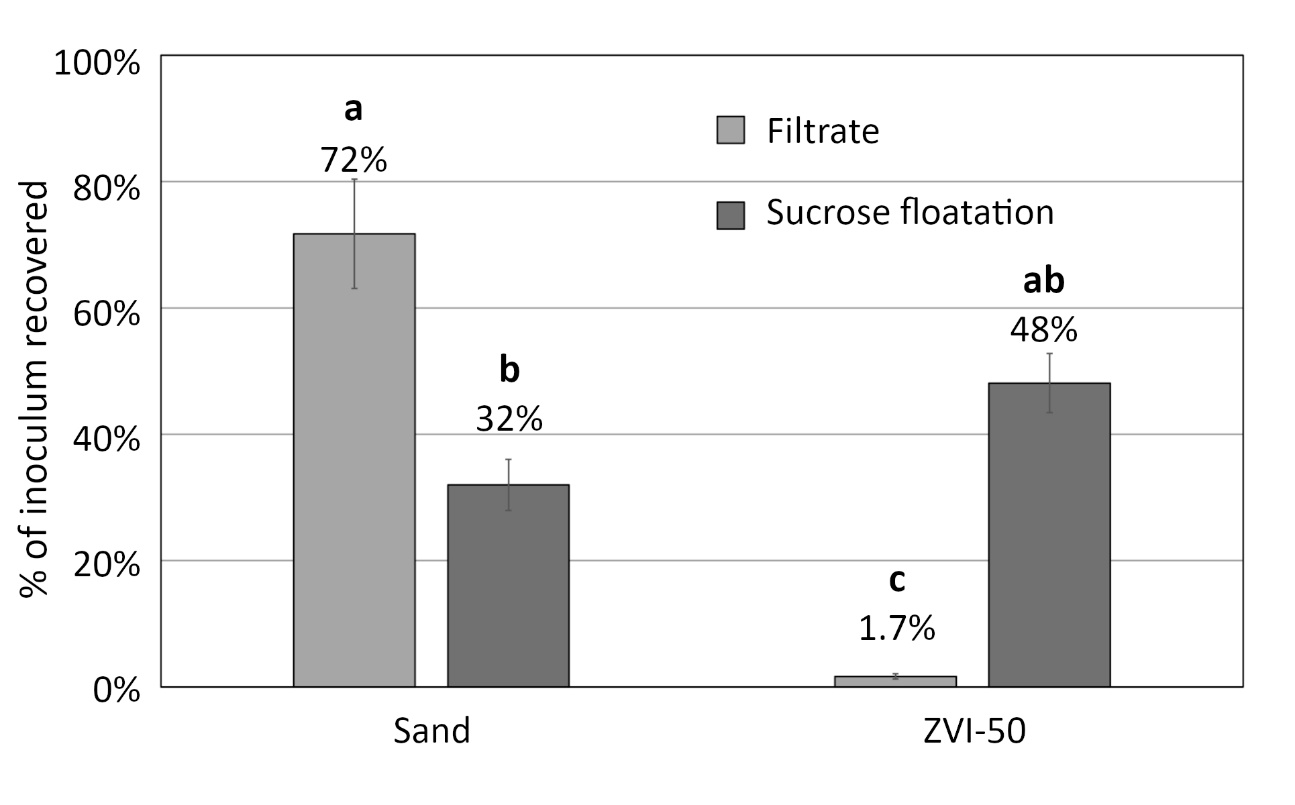


**Figure SD-2:** Recovery of *E. acervulina* from sand and ZVI-50 filters after filtration or sucrose floatation Error bars represent +/- 1 SD, bars with the same letter are not significantly different (ANOVA, 2-tailed t-test, *p*>0.05).
